# Approaches for classifying the indications for colonoscopy using detailed clinical data

**DOI:** 10.1186/1471-2407-14-95

**Published:** 2014-02-15

**Authors:** Hirut Fassil, Kenneth F Adams, Sheila Weinmann, V Paul Doria-Rose, Eric Johnson, Andrew E Williams, Douglas A Corley, Chyke A Doubeni

**Affiliations:** 1 University of Massachusetts Medical School, 50 Lake Ave North, Worcester, MA 01655, USA; 2 HealthPartners Institute for Education and Research, 8170 33rd Ave. S, Bloomington, MN 55425, USA; 3 Center for Health Research Northwest, Kaiser Permanente Northwest, 3800 N. Interstate Avenue, Portland, OR 97227, USA; 4 Division of Cancer Control and Population Sciences, National Cancer Institute, National Institutes of Health, 9609 Medical Center Dr., Room 3E438, Bethesda, MD 20892, USA; 5 Group Health Research Institute, 1730 Minor Ave #1600, Seattle, WA 98101, USA; 6 Center for Health Research Hawaii, Kaiser Permanente Hawaii, 501 Alakawa Street, Honolulu, HI 96817, USA; 7 Kaiser Permanente Division of Research, 2000 Broadway, Oakland, CA 94612, USA; 8 Department of Family Medicine and Community Health, and the Center for Clinical Epidemiology and Biostatistics at the Perelman School of Medicine, University of Pennsylvania, 222 Blockley Hall, 423 Guardian Drive, Philadelphia, PA 19104, USA; 9 The Center for Public Health Initiatives, University of Pennsylvania, Philadelphia, PA 19104, USA; 10 The Leonard Davis Institute of Health Economics, University of Pennsylvania, Philadelphia, PA 19104, USA

## Abstract

**Background:**

Accurate indication classification is critical for obtaining unbiased estimates of colonoscopy effectiveness and quality improvement efforts, but there is a dearth of published systematic classification approaches. The objective of this study was to evaluate the effects of data-source and adjudication on indication classification and on estimates of the effectiveness of screening colonoscopy on late-stage colorectal cancer diagnosis risk.

**Methods:**

This was an observational study in members of four U.S. health plans. Eligible persons (n = 1039) were age 55–85 and had been enrolled for 5 years or longer in their health plans during 2006–2008. Patients were selected based on late-stage colorectal cancer diagnosis in a case–control design; each case patient was matched to 1–2 controls by study site, age, sex, and health plan enrollment duration. Reasons for colonoscopies received in the 10-year period before the reference date were collected from three medical records sources (progress notes; referral notes; procedure reports) and categorized using an algorithm, with committee adjudication of some tests. We evaluated indication classification concordance before and after adjudication and used logistic regressions with the Wald Chi-square test to compare estimates of the effects of screening colonoscopy on late-stage colorectal cancer diagnosis risk for each of our data sources to the adjudicated indication.

**Results:**

Classification agreement between each data-source and adjudication was 78.8-94.0% (weighted kappa = 0.53-0.72); the highest agreement (weighted kappa = 0.86-0.88) was when information from all data sources was considered together. The choice of data-source influenced the association between screening colonoscopy and late-stage colorectal cancer diagnosis; estimates based on progress notes were closest to those based on the adjudicated indication (% difference in regression coefficients = 2.4%, p-value = 0.98), as compared to estimates from only referral notes (% difference in coefficients = 34.9%, p-value = 0.12) or procedure reports (% difference in coefficients = 27.4%, p-value = 0.23).

**Conclusion:**

There was no single gold-standard source of information in medical records. The estimates of colonoscopy effectiveness from progress notes alone were the closest to estimates using adjudicated indications. Thus, the details in the medical records are necessary for accurate indication classification.

## Background

There is a critical need for valid comparative effectiveness studies of cancer screening tests, but this is often hampered by uncertainties about the exact reason for testing. This is particularly important for observational studies that seek to determine the effectiveness of colorectal cancer (CRC) screening. There are multiple testing options available for CRC, [1,2] which differ in the strength of the evidence supporting their use, [3-11] and in their benefits, harms, costs, and complexity [3,12].

In the United States, colonoscopy is the most commonly used CRC screening test, [13] but it is also used in the diagnosis and surveillance of colorectal neoplasia [14]. Thus, the accurate determination and classification of the reasons for testing is crucial to the validity of observational studies of colonoscopy’s effectiveness and for guiding quality improvement efforts [15]. Further, the documented test indication, such as a prior diagnosis of adenoma or family history of CRC, guides clinicians in making follow-up recommendations [1,16,17]. However, there is currently a paucity of published studies on the process of using clinical data to assign indication.

The true indication for colonoscopy is the clinical rationale for the referral for testing, but this is difficult to measure from medical records or administrative data because the reasons for testing are not consistently documented [18]. Assigning an indication may also be difficult due to the multiplicity of reasons often recorded for a particular test or when common gastrointestinal symptoms, which have a low predictive value for CRC diagnosis, [19-21] are recorded at the time a colonoscopy is recommended or performed [15]. Therefore, colonoscopy indication derived from clinical or administrative data may be misclassified, leading to biased results in observational studies of screening colonoscopy effectiveness.

This study describes an algorithm and an adjudication approach for classifying colonoscopy indications using clinical data. We also determined the extent to which estimates of colonoscopy effectiveness based on pre-adjudication indication classification differed from an adjudicated reference standard by estimating the effect of screening colonoscopy on the risk of diagnosis with incident late-stage CRC.

The currently used approaches and published algorithms for assigning indication have not been validated against a standardized classification approach. Previous studies on classifying colonoscopy indication have simply been based on diagnosis and procedure codes in administrative or claims data that indicate the presence or absence of gastrointestinal-related procedures, signs, symptoms or conditions [22-25]. These algorithms can produce different classification results, depending on the codes used or the length of time prior to the test that was evaluated for ascertaining the presence or absence of gastrointestinal conditions. This can lead to unexpected results when evaluating the effectiveness of colonoscopy in observational data, [15,26] underscoring the need for a standardized approach for indication classification.

## Methods

The data were obtained from a case–control study of the comparative effectiveness of CRC screening tests [4]. Study patients were 55–85 years old between January 1, 2006 and December 31, 2008 and had been enrolled for ≥5 years in one of the following managed care plans: Group Health Cooperative, Washington State; Kaiser Permanente Hawaii; Kaiser Permanente Northwest; and Reliant Medical Group/Fallon Community Health Plan, Massachusetts. These health plans have used electronic medical records systems since at least 2005 and have electronic healthcare utilization data dating back to 1995 or earlier. This study was approved by the Institutional Review Boards at the University of Pennsylvania, the University of Massachusetts Medical School (UMMS), Group Health Research Institute (GHRI), and through ceded human subjects oversight authority from Reliant Medical Group to UMMS, and from Kaiser Permanente Hawaii and Kaiser Permanente Northwest to GHRI.

The outcome of the study was a diagnosis of incident late-stage CRC, defined as American Joint Commission on Cancer (Sixth Edition) stage IIB or higher based on tumor registry data [4,27]. Each patient with late-stage CRC (n = 498) was matched on the diagnosis (reference) date to 1–2 CRC-free controls (n = 541) by study site, birth year, sex, and health plan enrollment duration, as described elsewhere [4]. Data on the matching variables, socioeconomic factors, and patients’ clinical history were collected from electronic databases, tumor registry, and census data. Information on family history of CRC was obtained from electronic or paper medical records.

### Data collection on colonoscopy and other CRC tests

The primary interest in this report was the concordance of indication across multiple data sources for colonoscopies received during the 10-year period before the reference date (observation period), which was determined from data collected from each patient’s medical records (see Additional file 1: Appendix A). Trained abstractors, one each at three study sites and two at one site, performed the medical record audits. Audits were standardized through training and retraining and through the use of a common, structured electronic data collection instrument that was developed in Microsoft Access. The data collection tool was pre-populated with patient demographics, health care utilization history and the dates of CRC tests that were extracted from electronic databases using, in part, codes from the International Classification of Diseases, 9^th^ Edition, Clinical Modification, Current Procedural Terminology and Healthcare Common Procedure Coding System [28]. For each test found in the medical records, the auditors collected up to three documented reasons, separately, from each of three data sources (progress notes, referral note, and procedure report) according to 28 pre-coded categories (see Additional file 1: Appendix B). Auditors also collected reason-related information in free-text format. We defined the progress notes as all parts of the medical records other than the referral note and procedure-related documentation.

Similar data were collected on sigmoidoscopy, double contrast barium enema (BE), and CT colonography (CTC), which aided in indication classifications. Detailed data on fecal occult blood test (FOBT) restricted to the 5-year period before the reference date were also collected, including whether a test was positive or negative and the type of diagnostic test received following positive results. Auditors coded FOBT reasons as screening, diagnostic, surveillance, other, or unknown.

### Indication classification using a decision algorithm

**Figure 1:**
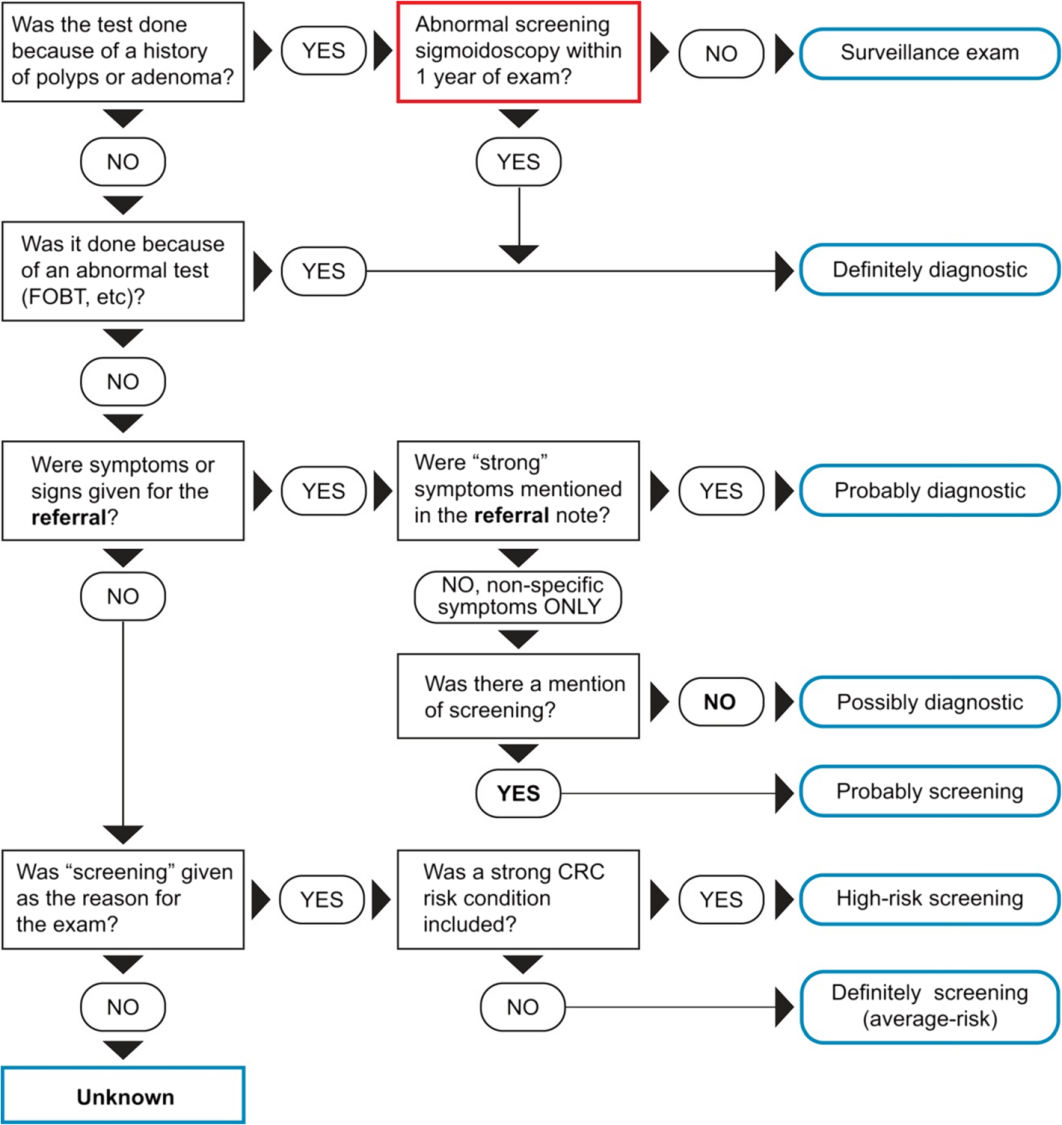
Decision algorithm for colonoscopy indication classification.

**Figure 2:**
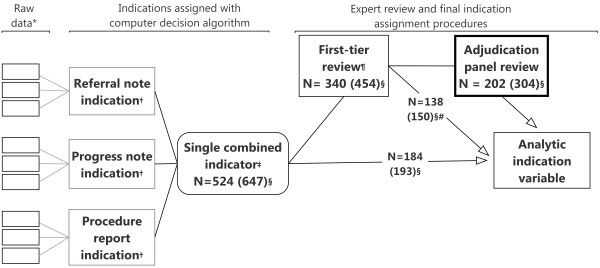
**Flow diagram of the derivation of indication variables for colonoscopy.** *Up to three coded reasons were recorded from each data source during the chart audit. †One indication variable was derived for each data source. ‡This is a single indication assigned to each test combining all coded data collected on each test during chart audit using the computer algorithm shown in Figure 1. It combined data from referral note, progress note and procedure report. §‘N’ is the number of patients. The numbers in parentheses are the tests received by the N patients. ¶A test was selected for review if more than one indication could be assigned or was unknown in all data sources, or relevant free-text data. #Tests on these patients were not selected for review and/or adjudication (see text).

We first used a computer-based decision algorithm to classify the indication for each colonoscopy test (test-level classification) into one of eight mutually exclusive categories: 1) surveillance, 2) ‘definite’ diagnostic, 3) ‘probable’ diagnostic, 4) ‘possible’ diagnostic, 5) ‘probable’ screening, 6) ‘definite’ average-risk screening, 7) ‘high-risk’ screening, or 8) unknown (Figure 1), followed by review of the classifications on selected tests (Figure 2). If a patient had multiple colonoscopies during the observation period, we derived a single overall indication variable to characterize his/her colonoscopy use (patient-level classification, described later).

A colonoscopy was classified as surveillance if performed for follow-up of previously detected polyps; ‘definite’ diagnostic if used to work-up a positive FOBT, a mass or other abnormal finding; ‘probable’ diagnostic if the medical records noted clinical conditions that were deemed to represent a high pretest probability for CRC, such as rectal bleeding; ‘possible’ diagnostic if the only documented reasons were non-specific medical conditions such as diarrhea or abdominal pain; or ‘probable’ screening if both non-specific symptoms and screening were recorded. The indication was considered ‘high-risk’ screening if the test was performed for screening and the patient had a first-degree relative diagnosed with CRC before age 50, two or more second-degree relatives diagnosed at any age, or other familial syndromes. The indication was considered ‘definite’ average-risk screening if screening was recorded and none of the CRC conditions or risk factors noted above were recorded. The indication was considered unknown if the reason was not specifically documented.

### Review of test indication

The algorithm assigned each test a single indication irrespective of the number of reasons (or missing data) recorded by chart auditors (see Figure 2). We therefore identified tests that could have been misclassified in order to review all available indication-related data. This review was conducted in two steps. The first step determined whether or not a particular test required a formal review by an adjudication panel of experts. Tests were selected for the first-tier review if more than one indication could be assigned, or indication was unknown in all data sources (Figure 2). For instance, a test was selected for review if the referral note recorded both constipation and average-risk screening or the indication differed (including unknown) across data sources (i.e., classified as ‘probable’ diagnostic based on referral note but ‘probable’ screening from progress notes). Because non-coded information was not included in the algorithm, we also reviewed all tests that had data in relevant free-text variables.

Three investigators and a research assistant (KA and see acknowledgement) performed the first-tier reviews of indication data (in pairs). At this review, tests that had additional pertinent indication-related information in free-text data or had substantive discordance across data sources were submitted for adjudication. Discordance due to classification as ‘definite’ diagnostic versus ‘probable’ diagnostic was considered non-substantive. We required consensus by both reviewers for a test to bypass adjudication. All tests classified as ‘high-risk’ screening were adjudicated to evaluate the details of the CRC risk. Once a test was selected for the first-tier review or adjudication, all the CRC tests of the particular patient (except FOBTs) were evaluated at the first-tier review, and/or adjudication, as appropriate. Of the 647 colonoscopies observed in the sample, 454 underwent the first-tier review of which 304 were reviewed by the adjudication panel (Figure 2).

### Adjudication of test indication

We formed a 5-member panel of experts comprised of epidemiologists, internists and gastroenterologists (DAC, VPDR and see acknowledgement), and a non-voting chair (CAD) to evaluate indication for the selected tests. The goal of adjudication was to classify each test according to the predetermined categories in Figure 1, after careful review of all available data. The adjudication committee reviewed tests blinded to the case–control status; study site; test type and exact dates; and, in the case of patient with multiple tests, whether a particular test was the trigger for adjudication. However, they were given the sequence and results of FOBTs and the sequence and type of health care visits.

In assigning indication, the committee considered clinical conditions that were documented as reasons for CRC testing, in part, by grouping them as strong versus non-specific based on the pretest probability of CRC associated with each condition (Additional file 1: Appendix C) [29,30]. Because gastrointestinal conditions are highly prevalent but are individually not highly predictive for CRC diagnosis [19,20,31], the grouping of clinical conditions was largely based on panel consensus. Disagreements among committee members on indication assignment were resolved using a majority rule. However, tests classified by different committee members as both screening and diagnostic were discussed until a consensus was reached.

### Assigning a single exposure variable per patient

Patients with multiple colonoscopies (n = 88) during the observation period were assigned a single patient-level indication in a temporally hierarchical manner by considering both the indication and the sequence of colonoscopies in relation to the reference date. We selected the ‘definite’ screening test with a test date that was farthest from the reference date; if none, then we used the earliest ‘probable’ screening colonoscopy; and if none, then ‘possible’ diagnostic, ‘probable’ diagnostic and finally ‘definite’ diagnostic colonoscopy, in that order. The indication was classified as surveillance if the first colonoscopy was for surveillance and there was no subsequent screening test.

### Statistical analyses

For this report, we categorized the indication as routine screening (‘probable’ or ‘definite’ average-risk screening), ‘high-risk’ screening, surveillance, ‘possible’ diagnostic, diagnostic (‘definite’ or ‘probable’ diagnostic), or unknown. Analyses were performed on both test-level (each colonoscopy, n = 647) and patient-level (n = 524) classifications. Pair-wise analyses compared the proportion classified in each of the six indication categories among data sources and with adjudication.

We calculated the percent concordance with adjudicated indication, for each data source individually and for all sources combined, in both test- and patient-level analyses. In these analyses, we considered all indication categories at the same time using a categorical variable, and combined routine and ‘high-risk’ screening into a single ‘screening’ category for ease of interpretation.

We also computed kappa (ĸ) coefficient of agreement using quadratic weights that considered the most important distinction as that between screening and diagnostic. The kappa statistic was interpreted according to Byrt’s recommendation (≤0.00 = no agreement; 0.01-0.20 = poor; 0.21-0.40 = slight; 0.41-0.60 = fair; 0.61-0.80 = good; 0.81-0.92 = very good; and >0.92 = excellent agreement) [32]. Kappa accounts for the probability of chance by considering both the observed and expected agreements. Thus, it can be spuriously low when expected agreement is high, as could occur in the case of indication classification due to high correlation among data sources. Therefore, we based our interpretation primarily on unweighted percentage concordance.

Next, we evaluated whether differences in the data sources and classification approach for indication influenced estimates of the association between exposure to routine screening colonoscopy and diagnosis with late-stage CRC. In secondary analyses, we used the expanded screening definition that included ‘high risk’ screening. Analyses were performed with conditional logistic regression models, adjusting for census block-group poverty levels (in quartiles), number of preventive health care visits, family history of CRC, modified Charlson comorbidity index at baseline, and receipt of other screening tests. We then computed the percentage difference in beta coefficients between the algorithm-derived screening indications and the adjudicated standard, and used two-sided Wald *χ*^2^*P*-values to evaluate the statistical significance of the differences. In our regression analyses, we accounted for the period of preclinical late-stage CRC by excluding tests performed within one month of the reference date, as described in a previous report [4]. The analyses were performed using STATA version 12.1 (StataCorp, College Station, TX, USA).

## Results

The patients (n = 1,039) included in this report were 72 years old on average, with an equal percentage of men and women (Table 1). Most had been members of their health plan for 10 years or longer. The majority of the colonoscopies received were for a diagnostic indication (59.4-69.7%), irrespective of the classification scheme or data source.

**Table 1:** Demographic and clinical characteristics of cases and controls, SEARCH Study 2006–2008, n = 1,039

**Characteristics**	**Sample, n = 1,039**
Age, year	
55-64	252 (24.3)
65-74	346 (33.3)
75-85	441 (42.4)
Female	515 (49.5)
Study site	
A	206 (19.8)
B	386 (37.2)
C	129 (12.4)
D	318 (30.6)
Poverty levels, quartiles*	
1	253 (24.4)
2	251 (24.2)
3	253 (24.4)
4	248 (23.9)
Missing	34 (3.3)
Length of enrollment with health plan before reference date, yr	
5.0-7.4	172 (16.6)
7.5-9.9	115 (11.1)
>10	752 (72.4)
Number of preventive outpatient health care visits within 5 years of reference date	
0	411 (39.6)
1	251 (24.2)
2-3	243 (23.4)
4+	134 (12.9)
Family history of colorectal cancer (CRC)^†^	96 (9.2)
Charlson comorbidity index at baseline‡	
0	842 (81.0)
1	148 (14.2)
2+	49 (4.7)
Had a healthcare visit during the 2-year period at baseline‡	161 (15.5)
Undergone colonoscopy	524 (50.4)
Had ≥2 colonoscopies	88 (8.5)

*Households below 1999 federal poverty levels within the block-group from 2000 decennial census measures. Higher quartiles correspond to higher levels of household poverty in the census block-group.

†This variable refers to family history that did not meet the exclusion – those with a history of colorectal cancer diagnosed in any first degree relative before age 50 or 2 or more relatives of any age, or other familial syndromes.

‡Baseline refers to the 2-year period at the beginning of each patient’s observation period.

**Figure 3:**
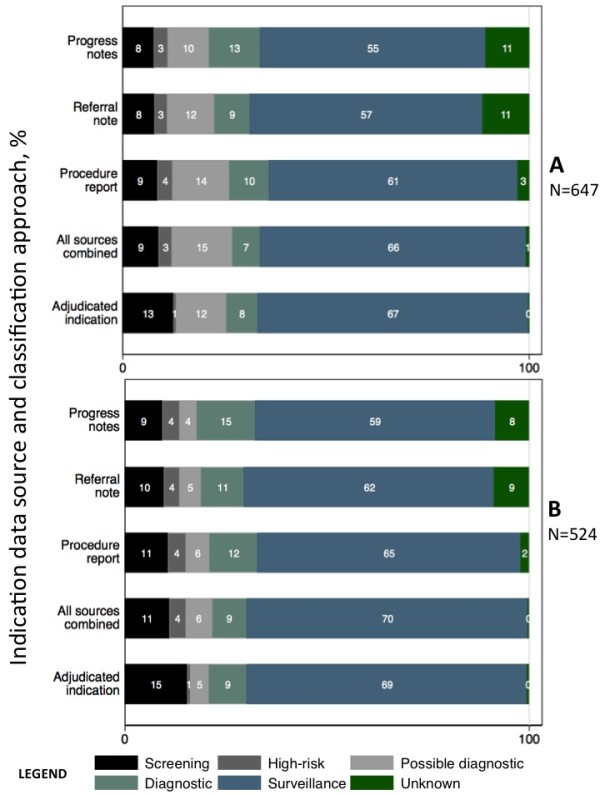
**Percentage distribution of colonoscopy indication by medical records data sources and targeted adjudication, at the test-level and analytic or patient-level.** *The numbers are the percentages in each classification group for colonoscopies in Figure 3A or patients in Figure 3B. There were 647 colonoscopies observed in 524 patients. The distribution of indication in Figure 3B, correspond to the analytic variable. Each of the colored sections of the stacked bars represents the classification of the indication as shown in the legend. The “all sources combined” indication is assigned with data from all sources using the classification algorithm.

The algorithm-based colonoscopy indication was categorized as ‘unknown’ for 2.8% of tests when based on the procedure report, 10.7% when based on the progress notes, and 11.4% when based on the referral note (Figure 3A). Compared to the procedure report, the progress note classified fewer tests as surveillance (13.9% versus 10.0%, P-value = 0.03). In patient-level analyses based on the algorithm-derived indications, a similar percentage of patients were classified as screening across the three data sources (progress note 9.4%, referral 9.7% and procedure report 10.7%) or ‘high-risk’ (Figure 3B).

### Indication classification after adjudication

The algorithm-based indications of the colonoscopies reviewed by the committee were: screening = 21, ‘high-risk’ = 21, surveillance = 80, ‘possible’ diagnostic = 8, diagnostic = 170, and unknown = 4 (Additional file 1: Appendix D). After the review, 16 (76.2%) indications previously classified as screening remained unchanged, but the remaining five were reclassified as ‘possible’ diagnostic (n = 2), diagnostic (n = 2) and surveillance (n = 1). Nineteen of the 21 ‘high-risk’ tests (90.5%), six of the 170 diagnostic (3.5%), one of the eight ‘possible’ diagnostic (12.5%) and two of the 80 surveillance tests (2.5%) were reclassified as screening. The majority of diagnostic tests (n = 155, 91.2%) remained unchanged; five were reclassified as ‘possible’ diagnostic, three as surveillance, and one as ‘high-risk’ screening. Only one of the four ‘unknowns’ remained unchanged, with one each of the remaining three reclassified as surveillance, ‘possible’ diagnostic and diagnostic.

### Indication classification agreement

**Figure 4:**
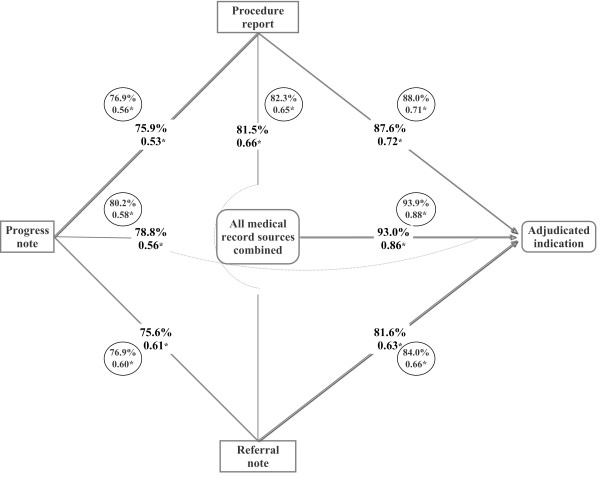
**Agreement on colonoscopy indication classification across three medical records data sources: test-level and patient-level analysis.** The percentages are the observed agreement and the proportions are the weighted kappa (ĸ) statistic. *The numbers in the circles are the patient-level analyses results.

Next, we analyzed agreement on classification across the indication categories. On individual colonoscopies (n = 647), the concordance on algorithm-based indication among the three data sources ranged from 75.6% (progress note versus referral) to 81.5% (procedure report versus referral), which corresponded to fair-good agreement on the kappa scale (ĸ = 0.53-0.66) (Figure 4). We also found fair-to-good agreement between adjudication and algorithm-based indication classification for each data source alone (78.8-87.6%, ĸ = 0.56-0.72), but very good agreement for all sources combined (93.0%, ĸ = 0.86).

In the patient-level analyses (n = 524), there was fair-to-good agreement in exposure classification among the three sources (76.9% to 82.3%, ĸ = 0.56-0.65) (Figure 4). Compared to adjudication, there was fair-to-good agreement with each of the data sources (progress note 80.2%, ĸ = 0.58; referral 84.0%, ĸ = 0.66; procedure report 88.0%, ĸ = 0.71); the highest level of agreement was with all sources combined (93.9%, ĸ = 0.88).

### Effect on relationship between screening colonoscopy and late-stage CRC

We then examined whether the differences in indication classification across data sources affected estimates of the effect of screening. We estimated effects of screening colonoscopy on incident late-stage CRC diagnosis risk, comparing algorithm-derived screening indications to adjudication, according to the source of indication data. We found that the associations of screening colonoscopy with late-stage CRC diagnosis risk differed from the adjudicated standard by 2.4-34.9% (Table 2). The estimates based on progress note information alone (P-values = 0.64-0.98) or in combination with the other two sources (P-values = 0.52-0.69) showed relatively little difference from adjudication. The estimates for the effects of screening colonoscopy on late-stage CRC based on analyses with information from the referral (P-values = 0.12-0.41) or procedure report (P-values = 0.23-0.26) showed slightly more deviation from the adjudicated standard than progress notes (see Table 2).

**Table 2:** Association between screening colonoscopy and risk of incident late-stage CRC according to data source, SEARCH Study 2006–2008, n = 1,039

**Data source according to screening definition used**	**Odds Ratio and 95% CI**	**% Difference in beta coefficients**	P-value of difference*
*Screening defined as ‘probable’ or ‘definite’*			
Progress note	0.31 (0.14-0.70)	−9.5	0.64
Referral note	0.46 (0.22-0.98)	28.0	0.41
Procedure report	0.50 (0.25-1.02)	31.2	0.26
All sources combined	0.30 (0.14-0.65)	−21.6	0.52
Adjudicated indication	0.36 (0.19-0.68)	Ref	Ref
*Same definition as above plus ‘high-risk’ screening exposures*			
Progress note	0.32 (0.17-0.64)	2.4	0.98
Referral note	0.45 (0.23-0.85)	34.9	0.12
Procedure report	0.43 (0.23-0.79)	27.4	0.23
All sources combined	0.31 (0.16-0.58)	−6.2	0.69
Adjudicated indication	0.33 (0.17-0.62)	Ref	Ref

Conditional logistic regression modelling was performed with separate indicator variables for colonoscopy and sigmoidoscopy and adjusted for receipt of ‘definite’ screening barium enema and FOBT, census block-group poverty levels (as a continuous variable), number of preventive health care visits, family history of colorectal cancer, and comorbidity index at baseline. Missing values of poverty level were imputed using predictive mean matching.

*Two-sided Wald Chi-square (*χ*^2^) P-values of the difference between effect sizes.

## Discussion

This study compared the information from different clinical data sources for colonoscopy indication classification and found generally good agreement among the progress notes, referral note, and procedure report. However, there were differences between sources in the classification of tests as screening and the extent of missing information. After adjudication, most patients classified as ‘high-risk’ were determined to be average-risk screening. Indication classification without expert review resulted in a 2.4-34.9% deviation from the adjudicated standard in the estimated effects of screening colonoscopy. We found that, although the direction of the association between screening colonoscopy and late-stage CRC diagnosis risk was not changed by the indication data source, analyses with information from the progress notes alone or in combination with referral and procedure reports produced results that were closest to those from the indication derived through adjudication.

The literature provides no consistent method for determining CRC test indication and no previously published studies have described the use of adjudication in systematically assigning indication. Most reports using medical records derive indication from the procedure report alone and in some cases the source of the indication information in the medical records was not clearly described [18,33-36]. Our findings suggest that approaches using only the procedure report or referral notes may be subject to a greater degree of misclassification, possibly because the indication documented may be influenced by examination findings or the need to obtain third-party payer approval for the referral.

Our study has several important implications. First, compared with adjudication, all of the sources of information demonstrated some misclassification, particularly for ‘high-risk’ indications. Second, the procedure report had the fewest missing indications, but produced effect sizes that differed slightly more from the adjudicated results than the progress notes. Third, the progress notes data produced estimates of screening that were consistently closest to those from adjudication, suggesting that the details from progress notes are important for accurate indication classification. Thus, our study suggests that review of data in the progress notes in medical records, including detailed information on clinical conditions documented around the time of the test, is required to produce valid results in observational studies of CRC screening effectiveness. Finally, if resources are limited, adjudication of indication may focus on ‘high-risk’ and ‘unknown’ test indications. If adjudication is not performed, given their relative rarity, including ‘high-risk’ indication as screening is preferable to excluding them in analyses of effects on average-risk persons.

This study has some limitations. Because the original study was for average-risk persons, some high-risk patients were excluded at the time of patient selection. Therefore, tests for high-risk indications may be underrepresented in this analysis. Abstractors were not blinded to the source of information in the medical records, possibly contributing to the high correlation of indication across data sources. Also, not all tests were adjudicated, and reviewers did not have access to all the medical record data, including detailed information on the duration and severity of clinical conditions that were recorded as reasons for testing. Further, the distribution of colonoscopy indications, and thus the usefulness and necessary extent of adjudication, may vary across settings, depending on population demographics and reimbursement policies. Future larger studies in non-managed care settings and in different settings or populations are needed to establish the benefits of obtaining data from multiple sources and conducting adjudication for indication classification. Additional studies are also needed to evaluate the impact of indication misclassification on estimates of the effectiveness of colonoscopy for reducing risk of CRC death. Further, the approaches described in this paper can be applied to evaluate the degree to which indication misclassification biases results of colonoscopy effectiveness in studies based on administrative data.

## Conclusions

Careful classification of indication is important in observational research on the comparative effectiveness of CRC screening tests and in the quality improvement of CRC testing. In our study, we found no single gold-standard source of information in the medical records for indication classification that agreed consistently with expert adjudication, and the data sources were complementary in achieving better indication classification. Adjudication changed the classification of some indications and the data-source differences we observed resulted in some deviations in the odds ratios for the association between screening colonoscopy and late-stage CRC risk. The deviations from the adjudicated standard for this association were smaller with progress notes information than with other sources alone. Therefore, careful standardized reviews of information in the progress notes, referral notes and procedure report are necessary for accurate classification of colonoscopy indication.

## Competing interests

The authors declare that they have no competing interests.

## Authors’ contributions

HF participated in drafting the manuscript and critical revision of the manuscript. CAD was PI of the study, conceptualized the study, conducted the analyses, and drafted the manuscript. KA, SW, AW, VPDR and DAC participated in study conceptualization, and critical revision of the manuscript. EJ participated in revisions to the manuscripts. All authors read and approved the final manuscript.

## Pre-publication history

The pre-publication history for this paper can be accessed here:

http://www.biomedcentral.com/1471-2407/14/95/prepub

## Supplementary Material

Additional file 1: Appendix A Data elements and sources for medical records audits. **Appendix B.** The 28 pre-coded indication categories used for medical records audits. **Appendix C.** Classification of clinical conditions for colonoscopy indication adjudication according to pretest probability of colorectal cancer diagnosis. **Appendix D.** Table of distribution of indication classifications before and after adjudication for tests that underwent panel review. Click here for file
