# Supplementary material for: Approaches for classifying the indications for colonoscopy using detailed clinical data
Source: BMC Cancer. 2014 Feb 15;14:95. doi: 10.1186/1471-2407-14-95 (PMC3927818; doi:10.1186/1471-2407-14-95)
Supplement: Additional file 1: Appendix A — Data elements and sources for medical records audits. Appendix B. The 28 pre-coded indication categories used for medical records audits. Appendix C. Classification of clinical conditions for colonoscopy indication adjudication according to pretest probability of colorectal cancer diagnosis. Appendix D. Table of distribution of indication classifications before and after adjudication for tests that underwent panel review. [file 1471-2407-14-95-S1.docx]

# Additional file 1 – APPENDICES A-D

### Appendix A – Data elements and sources for medical records audits

| ***Data elements*** | ***Data source**** |
| --- | --- |
| Demographics |  |
| Age, sex, and race/ethnicity | Tumor registry, and administrative data* |
| Outpatient health care visit history |  |
| Date of earliest medical visit after age 30 | Electronic health care utilization data* |
| Number of routine wellness examinations |  |
| Date of last physical examination |  |
| Health plan enrolment duration | Administrative data |
| Initial eligibility determination |  |
| Family history of colorectal cancer and other familial syndromes^†^ | Electronic data, and medical records audits |
| Patient’s history of colorectal cancer | Tumor registry |
| Inflammatory bowel disease  Colectomy | Electronic data on diagnoses and procedures* |
| Colorectal cancer (CRC) testing |  |
| Number of CRC tests found | Electronic data on procedures, and laboratory databases* |
| Discussion and ordering of CRC tests | Medical record audits |
| Fecal occult blood testing ‡ |  |
| Date ordered, collected, or performed | Electronic data on procedures, laboratory databases, and medical record audits |
| Reason for test |  |
| Result of test |  |
| If positive, diagnostic test ordered |  |
| Colorectal cancer tests (procedures) |  |
| Type and date of test | Electronic data on procedure* |
| Reason for test | Medical record audits |
| Complications of the test |  |
| Specialty of provider performing the test |  |
| FOBT performed in the period prior to a test |  |
| Quality of the bowel preparation (poor to excellent; adequacy, and other descriptors) |  |
| Polyps or lesions found |  |
| Completeness of test§ |  |
| Total duration of test, and withdrawal time§ |  |

**Note**: All data were collected from the 10-year period before the reference date.

*Data from electronic databases were confirmed during medical record audits.

†Details on familial syndromes were collected from medical records in the two-year period before the reference date.

‡Details on fecal occult blood test were restricted to the 5-year period before the reference date.

§These were not collected on barium enema tests

### Appendix B – The 28 pre-coded indication categories used for medical records audits:

1. Positive stool blood test
2. Abnormal sigmoidoscopy
3. Abnormal barium enema
4. Abnormal imaging (X-ray, CT scan, etc.)
5. Abdominal pain
6. Abnormal weight loss
7. Abdominal mass
8. Anemia, iron deficiency type
9. Other types of anemia
10. Rectal bleeding
11. Melena
12. Other GI bleeding
13. Change in bowel habits
14. Constipation
15. Suspected colon cancer
16. Unspecified colitis
17. Crohn’s or ulcerative colitis
18. Diarrhea
19. Family history of colon cancer
20. Familial adenomatous polyposis or LYNCH
21. Other familial syndromes
22. Irritable bowel syndrome
23. Metastatic cancer work-up
24. Colon/rectal polyps or adenomas
25. Screening (routine)
26. Therapeutic
27. Other specified reasons
28. No other information was documented

### Appendix C – Classification of clinical conditions for colonoscopy indication adjudication according to pretest probability of colorectal cancer diagnosis

| **Strong symptoms (strongly associated)** | **Non-specific symptoms** |
| --- | --- |
| Acute bowel obstruction  Perforation  Abdominal mass  Massive lower GI bleeding  Bleeding  Bright red blood per rectum  Iron deficiency anemia  Elevated CEA  Suspected IBD | Weight loss  Abdominal pain or fullness  Constipation  Change in bowel habits, diarrhea, or altered stools  Rectal pain  Fatigue  Other anemias  Nausea and vomiting  Anorexia  Other non-specific abdominal symptoms |

### Appendix D – Table of distribution of indication classifications before and after adjudication for tests that underwent panel review

| Data sources and Indication type, n(%) | Indication | | | | | |
| --- | --- | --- | --- | --- | --- | --- |
|  | Screening | High risk | Surveillance | Possible diagnostic | Diagnostic | Unknown |
| **Test-level classification, n=304** |  |  |  |  |  |  |
| Progress note | 18 (5.9) | 22 (7.2) | 53 (17.4) | 37 (12.2) | 137 (45.1) | 37 (12.2) |
| Referral note | 16 (5.3) | 20 (6.6) | 63 (20.7) | 20 (6.6) | 139 (45.7) | 46 (15.1) |
| Procedure report | 19 (6.3) | 24 (7.9) | 76 (25.0) | 23 (7.6) | 150 (49.3) | 12 (4.0) |
| All three sources combined | 21 (6.9) | 21 (6.9) | 80 (26.3) | 8 (2.6) | 170 (55.9) | 4 (1.3) |
| Adjudicated indication | 44 (14.5) | 4 (1.3) | 65 (21.4) | 13 (4.3) | 177 (58.2) | 1 (0.3) |
| **Patient level classification, n=202** |  |  |  |  |  |  |
| Progress note | 17 (8.4) | 22 (10.9) | 15 (7.4) | 31 (15.4) | 103 (51.0) | 14 (6.9) |
| Referral note | 16 (7.9) | 20 (9.9) | 20 (9.9) | 19 (9.4) | 107 (53.0) | 20 (9.9) |
| Procedure report | 19 (9.4) | 23 (11.4) | 23 (11.4) | 22 (10.9) | 110 (54.5) | 5 (2.5) |
| All three sources combined | 21 (10.4) | 21 (10.4) | 25 (12.4) | 8 (4.0) | 126 (62.4) | 1 (0.5) |
| Adjudicated indication | 44 (21.8) | 4 (2.0) | 16 (7.9) | 12 (5.9) | 125 (61.9) | 1 (0.5) |
